# Life’s Essential 8 is inversely associated with high-sensitivity C-reactive protein

**DOI:** 10.1038/s41598-024-65977-3

**Published:** 2024-07-01

**Authors:** Lana Hebib, Ángel Herraiz-Adillo, Sara Higueras-Fresnillo, Daniel Berglind, Bledar Daka, Patrik Wennberg, Emil Hagström, Cecilia Lenander, Viktor H. Ahlqvist, Carl Johan Östgren, Karin Rådholm, Pontus Henriksson

**Affiliations:** 1 Department of Health, Medicine and Caring Sciences, Linköping University, Linköping, Sweden; 2 Department Physical Education, Sport and Human Motricity, Universidad Autónoma de Madrid, Madrid, Spain; 3 Department of Global Public Health, Karolinska Institutet, Stockholm, Sweden; 4 Centre for Epidemiology and Community Medicine, Region Stockholm, Stockholm, Sweden; 5 School of Public Health and Community Medicine, Institute of Medicine, Sahlgrenska Academy, University of Gothenburg, Gothenburg, Sweden; 6 Department of Public Health and Clinical Medicine, Umeå University, Umeå, Sweden; 7 Department of Medical Sciences, Cardiology, Uppsala University, Uppsala, Sweden; 8 Department of Clinical Sciences in Malmö, Centre for Primary Health Care Research, Lund University, Lund, Sweden; 9 Department of Biomedicine, Aarhus University, Aarhus, Denmark; 10 Institute of Environmental Medicine, Karolinska Institutet, Stockholm, Sweden; 11 Centre for Medical Image Science and Visualization (CMIV), Linköping University, Linköping, Sweden; 12 The George Institute for Global Health, University of New South Wales, Sydney, Australia

**Keywords:** Biomarkers, Cardiology, Medical research, Pathogenesis, Risk factors

## Abstract

Life’s Essential 8 (LE8) is a score that includes modifiable risk factors for cardiovascular disease. Four health behaviors (diet, physical activity, nicotine exposure and sleep health) and four health factors (non-HDL cholesterol, blood glucose, blood pressure and body mass index) are included. These modifiable risk factors promote inflammation, and inflammation is one of the biological mechanisms of cardiovascular disease development. Thus, we examined the relationship between cardiovascular health measured by LE8 and low-grade inflammation measured by high-sensitivity C-reactive protein (hs-CRP) in the cross-sectional population-based Swedish CArdioPulmonary bioImage Study (SCAPIS). The study consisted of 28,010 participants between 50 and 64 years (51.5% women, mean age 57.5 years). All individual LE8 components were assigned a score between 0 (unhealthy) and 100 (healthy) points, and a global score was calculated. The association between LE8 scores and high-risk hs-CRP (defined as > 3.0 mg/L) was analyzed using adjusted logistic regression with spline analyses. There was a strong, dose response and inverse association between LE8 scores and levels of hs-CRP. Thus, those with a low LE8 score (= 50.0 points) had 5.8 higher (95% confidence interval [CI] 5.2–6.4) odds ratio (OR) of having high hs-CRP as compared to those with a high LE8 score (= 80.0 points). In conclusion, our findings show strong inverse associations between LE8 scores and levels of hs-CRP.

## Introduction

Inflammation is suggested to play an important role in the onset and development of cardiovascular disease^1–3^. C-reactive protein (CRP) is an inflammatory marker of atherothrombosis^4^, but it has been suggested that CRP is not a causal factor in coronary heart disease^5,6^. However, elevated levels of high-sensitivity C-reactive protein (hs-CRP) have been shown to predict all-cause and cardiovascular mortality independently of traditional risk factors^7,8^, indicating its potential as a biomarker. A hs-CRP > 3.0 mg/L is associated with a greater risk of cardiovascular disease^9^, while values of hs-CRP > 10.0 mg/L are often caused by other conditions such as infectious diseases, trauma and autoimmune disorders. Nevertheless, chronic elevations above 10.0 mg/L are also associated with higher risks of cardiovascular disease^9^.

Less is known about the extent to which traditional cardiovascular risk factors correlate with hs-CRP. Cardiovascular risk may be assessed using the concept Ideal Cardiovascular Health (CVH) and Life’s Simple 7 (LS7), established by the American Heart Association (AHA) in 2010 in an effort to improve cardiovascular health in the United States population and highlighting the low prevalence of ideal CVH^10^. The construct was updated in 2022 and is now well established and supported by previous research^11–14^. The updated concept, Life’s Essential 8 (LE8), includes a new metric, sleep health, in addition to the seven metrics used in LS7. LE8 consists of health behaviors (diet, physical activity, nicotine exposure and sleep duration) as well as health factors (body mass index [BMI], non-HDL cholesterol, fasting blood glucose and blood pressure)^11^. The scoring system in LE8 has been updated as well, and is now ranging from 0 (lowest CVH) to 100 points (highest CVH)^11^. Recent studies have shown that a low LE8 score is strongly associated with higher risks of coronary and carotid atherosclerosis^15,16^ as well as cardiovascular and all-cause mortality^11–14^.

To the best of our knowledge, the association between LE8 and inflammation has not been evaluated in any previous study. However, previous studies have suggested that CVH assessed by LS7 is associated with hs-CRP^17–22^. Of these studies, only one was done in Europeans^17^, and it only included men, which makes generalization to women impossible^17^. Therefore, the aim of this study was to investigate if CVH measured by LE8 is associated with subclinical inflammation (as measured by hs-CRP) in a general population sample.

## Methods

### Study design and population

The Swedish CArdioPulmonary bioImage Study (SCAPIS) is a multicenter general population-based study conducted between 2013 and 2018 aiming at predicting and preventing cardiovascular and pulmonary disease. Participants were randomly invited from the Swedish population register and the study includes 30,154 men and women aged between 50 and 64 years. The overall participation rate was 50.3% and was similar between men and women as well as age groups. The six study sites (Gothenburg, Linköping, Malmö/Lund, Stockholm, Umeå and Uppsala) recruited around 5000 participants from each municipality area. Extensive examinations were performed at the respective university hospitals. The study design is described in detail in the study protocol^23^.

**Figure 1:**
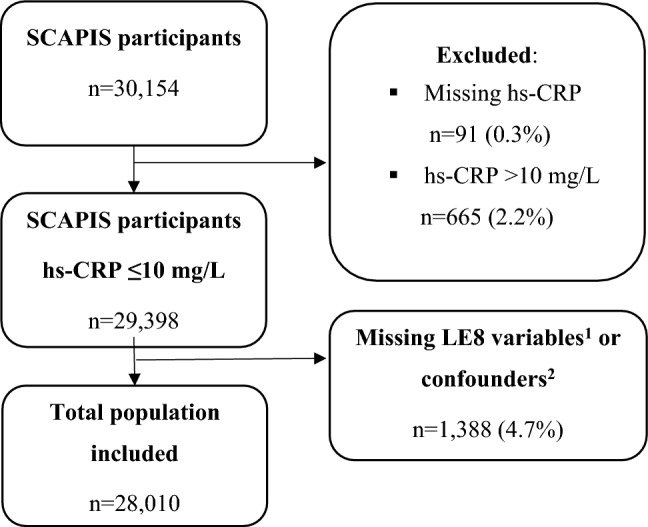
Flow chart of the analytic sample. Of 30,154 participants, 91 lacked data of hs-CRP. A total of 665 participants had hs-CRP > 10 mg/L and another 1,388 had no data of LE8 or the confounding factors. Thus, the final analytic sample comprised of 28,010 participants. ^1^Diet, physical activity, nicotine exposure, sleep duration, body mass index, non-HDL cholesterol, fasting glucose and blood pressure. ^2^Age, sex, study center, education, and alcohol use. hs-CRP: high-sensitivity C-reactive protein, LE8: Life’s Essential 8 score, SCAPIS: Swedish CArdioPulmonary bioImage Study.

Figure 1 presents a flow chart of the study. Of the 30,154 participants, 91 lacked data of hs-CRP. An additional 665 participants had hs-CRP > 10.0, which may indicate inflammatory processes such as an ongoing infection and were therefore excluded. Finally, a total of 1,388 participants had no data of LE8 or the covariates. Thus, the final analytic sample consisted of 28,010 participants.

Informed consent was obtained from each participant. All methods were performed in accordance with the relevant guidelines and regulations. The study has been approved by the Swedish Ethical Review Authority (reference numbers: 2021-06408-01 and 2022-04375-02).

### Life’s Essential 8 and covariates

The measurements and categorization of the LE8 metrics have been described in detail previously^15^. Diet intake was reported in the web-based and validated questionnaire MiniMeal-Q and scores were calculated considering the Mediterranean Eating Pattern for Americans^24^.

Physical activity was measured during 7 days with tri-axial accelerometry (Actigraph^®^ GT3X+, wGT3X+ and wGT3X-BT). Nicotine exposure and sleep health were assessed by self-reported questionnaires. Weight was measured on a balance beam or digital scale, while height was measured to the nearest centimeter in standing position with legs kept together and spine in an upright position. BMI was later calculated as kg/m^2^. Non-HDL cholesterol and glucose levels were measured in venous plasma samples after overnight fasting (Architect Abbott^®^ and Cobas Roche^®^). Fasting capillary glucose values (HemoCue^®^ Glucose 201RT Systems) were used to estimate venous plasma glucose (IFCC standard) in the 2,703 participants with missing venous plasma samples^25^. Fasting venous blood samples were used to analyze HbA1c which reflect glucose levels during the past 2 to 8 weeks. In addition, the use of antidiabetic medication was considered, and participants taking any antidiabetic medication were categorized as having diabetes. Brachial arterial blood pressure was measured in supine position with an automated oscillometric device (OMRON M10-IT, Omron Healthcare Co. Ltd, Japan) after 5 min of rest. A comprehensive questionnaire was used to obtain data and characterize the participants regarding the covariates age, sex, study center, alcohol use and education levels^23^.

Each of the eight metrics was assigned a score ranging from 0 to 100 as described in detail previously^15^. The highest scores (i.e., 100) were assigned to an ideal diet, ≥ 150 min of at least moderate-intensity activity per week, no history of smoking, an average of 7– < 9 h of sleep per night, BMI < 25 kg/m^2^, untreated non–HDL cholesterol < 130 mg/dL, no history of diabetes and untreated fasting blood glucose < 100 mg/dL (or HbA1c < 5.7%) and untreated blood pressure < 120/ < 80 mm Hg. The lowest scores (i.e., 0) were assigned to the least ideal diet, 0 min of at least moderate-intensity activity per week, current smokers, an average of < 4 h of sleep per night, BMI ≥ 40.0 kg/m^2^, non–HDL cholesterol ≥ 220 mg/dL, diabetes with HbA1c ≥ 10.0% and blood pressure ≥ 160 or ≥ 100 mmHg, in systolic and diastolic values, respectively.

Participants were only included in the study if they had data of at least seven of the eight metrics. A global LE8 score from 0 to 100 was calculated as the unweighted average of all of the present metrics in accordance to the AHA guideline^11^. A behavioral score was calculated as the average of diet, physical activity, nicotine exposure and sleep health scores. The factor score was also calculated as the average of body mass index, non-HDL cholesterol, glucose and blood pressure scores.

The LE8 variable was considered as continuous and further categorized, in accordance with the American Heart Association, as low CVH (0–49 points), intermediate CVH (50–79 points) and high CVH (80–100 points).

### High-sensitivity C-reactive protein

The hs-CRP levels were measured after overnight fast (Architect Abbott^®^ and Cobas Roche^®^). Due to variations in the low detection limits across different study sites, the lowest detection limit was harmonized to 0.6 mg/L across all sites in the general sample. Values ≤ 3.0 mg/L were classified as low-risk whereas values that were between 3.1 and 10.0 mg/L were classified as high-risk hs-CRP, consistent with previous studies^18,19,22^. Values > 10.0 mg/L (2.2%) were excluded in the analysis.

### Statistical analyses

A complete case analysis was conducted, excluding those with missing data on hs-CRP, LE8 variables or confounders (N = 2144, 7.2% excluded). Continuous variables were presented as means with standard deviations and categorical variables were presented as frequencies with percentages.

The adjusted continuous non-linear relationships between the LE8 score and high-risk hs-CRP were estimated using logistic regression with restricted cubic splines of the LE8 distribution, with four knots at the 5th, 35th, 65th, and 95th percentiles^26^. A LE8 score of 80.0 points was used as the reference category. Secondly, the association between LE8 and high-risk hs-CRP was analyzed using binary logistic regression models, depicting odds ratios (OR) and 95% confidence intervals (CI). Two models were used: (1) an unadjusted model, and (2) a model adjusted for age, sex, study center, education level, and alcohol use.

In addition to the analysis with continuous LE8 data, an additional analysis was conducted examining associations of categories of LE8 scores (≤ 49 points, 50–79 points, ≥ 80 points) with hs-CRP using binary logistic regression. To examine the robustness of the main findings and in consonance with a previous SCAPIS publication^27^, sensitivity analyses were performed to obtain OR for the association between CVH and high-risk hs-CRP by using a more conservative cut-off for defining ideal physical activity (300 min of moderate -or greater- intensity activity per week instead of 150 min per week). This was done due to the high prevalence of ideal physical activity in our study which may reflect an overestimation by the accelerometry. In addition, the association between CVH and high-risk hs-CRP, including those (n = 665) with hs-CRP > 10 mg/L was estimated as well as incorporating additional adjustments for economic status and history of cardiovascular disease. The association of the individual components of LE8 and hs-CRP was analyzed by calculating Z-scores which shows the relative importance of the individual components of LE8.

P-values < 0.05 were considered statistically significant. IBM SPSS (Armonk, NY: IBM Corp) statistics version 27 and STATA 17 (StataCorp. 2021) were used to perform all statistical analyses.

## Results

### Characteristics of the study population

Table 1 depicts the characteristics of the study population. Of the 28,010 participants, 14,417 (51.5%) were women and the mean age was 57.5 years. On average, blood pressure was 125.8 ± 17.0/77.5 ± 10.5 mmHg, and physical activity, sleep and HbA1c were at recommended levels (56.2 ± 29.8 min/day, 89.5% slept 6–9 h per night and 36.4 ± 6.2 mmol/mol, respectively). Meanwhile, average non-HDL cholesterol and BMI were above goal levels (3.4 ± 1.0 mmol/L and 26.9 ± 4.4 kg/m^2^, respectively). Furthermore, the mean diet score was low (8.2 ± 2.2 points in the Mediterranean Eating Pattern for Americans Questionnaire). Less than 50% of participants were current or previous smokers. Regarding LE8 score, 3.9%, 72.2% and 23.9% of the population had scores below 50, between 50 and 79, and of 80 and above, respectively. As many as 30.8% of women had high global CVH (defined as an LE8 score ≥ 80) while only 16.5% of men had the same status (p < 0.001). Finally, 14.4% of participants had high-risk levels of hs-CRP (> 3.0 mg/L). Results stratified by sex showed that 15.7% of women had high-risk levels while 13.0% of men had these levels (p < 0.001).

**Table 1:** Characteristics of the study population.

Characteristic	Total (n = 28,010)	Men (n = 13,593)	Women (n = 14,417)
Age, years	57.5 ± 4.3	57.5 ± 4.4	57.5 ± 4.3
Highest completed level of education			
Not completed primary school	171 (0.6%)	86 (0.6%)	85 (0.6%)
Primary school	2380 (8.5%)	1321 (9.7%)	1059 (7.3%)
High school	12,727 (45.4%)	6614 (48.7%)	6113 (42.4%)
College or university	12,732 (45.5%)	5572 (41.0%)	7160 (49.7%)
Alcohol use			
Never	2479 (8.9%)	999 (7.3%)	1480 (10.3%)
Monthly or less	4300 (15.4%)	1746 (12.8%)	2554 (17.7%)
2–4 times a month	10,739 (38.3%)	5220 (38.4%)	5519 (38.3%)
2–3 times a week	8454 (30.2%)	4322 (31.8%)	4132 (28.7%)
≥ 4 times a week	2038 (7.3%)	1306 (9.6%)	732 (5.1%)
hs-CRP	1.7 ± 1.7	1.6 ± 1.6	1.7 ± 1.7
≤ 3.0 mg/L	23,973 (85.6%)	11,824 (86.7%)	12,149 (84.3%)
> 3–10^a^ mg/L	4037 (14.4%)	1769 (13.0%)	2268 (15.7%)
Behaviors			
Diet, points in MEPA Questionnaire	8.2 ± 2.2	7.7 ± 2.0	8.8 ± 2.1
Physical activity, min/day	56.2 ± 29.8	58.2 ± 31.5	54.3 ± 28.0
Current smoker	3447 (12.3%)	1664 (12.2%)	1783 (12.4%)
Former smoker	10,105 (36.1%)	4545 (33.4%)	5560 (38.6%)
Never smoker	14,170 (50.6%)	7256 (53.4%)	6914 (48.0%)
Sleep, ≤ 4–5 h/night	2662 (9.6%)	1182 (8.8%)	1480 (10.3%)
Sleep, 6–9 h/night	24,842 (89.5%)	12,192 (90.5%)	12,650 (88.4%)
Sleep, ≥ 10 h/night	173 (0.6%)	68 (0.5%)	105 (0.7%)
Health factors			
Body mass index, kg/m^2^	26.9 ± 4.4	27.4 ± 3.9	26.4 ± 4.7
Non-HDL cholesterol, mmol/L	3.4 ± 1.0	3.4 ± 1.0	3.4 ± 1.0
HbA1c, mmol/mol	36.4 ± 6.2	36.7 ± 7.0	36.2 ± 5.4
Systolic blood pressure, mmHg	125.8 ± 17.0	128.7 ± 15.6	123.0 ± 17.8
Diastolic blood pressure, mmHg	77.5 ± 10.5	78.4 ± 10.1	76.6 ± 10.8
LE8 score, 0–100	70.8 ± 11.5	68.6 ± 11.1	72.9 ± 11.6
≤ 49	1103 (3.9%)	662 (4.9%)	441 (3.1%)
50–79	20,224 (72.2%)	10,691 (78.7%)	9533 (66.1%)
≥ 80	6683 (23.9%)	2240 (16.5%)	4443 (30.8%)

Quantitative variables are depicted as mean ± SD.

Categorical variables are depicted as frequencies (percentages).

HbA1c: Hemoglobin A1c, HDL: high-density lipoprotein, hs-CRP: high-sensitivity C-reactive protein, LE8: Life's Essential 8, MEPA: Mediterranean Eating Pattern for Americans, SD: standard deviation.

^a^Participants with hs-CRP > 10 mg/L were excluded (n = 665).

### Associations between LE8 and hs-CRP

**Figure 2:**
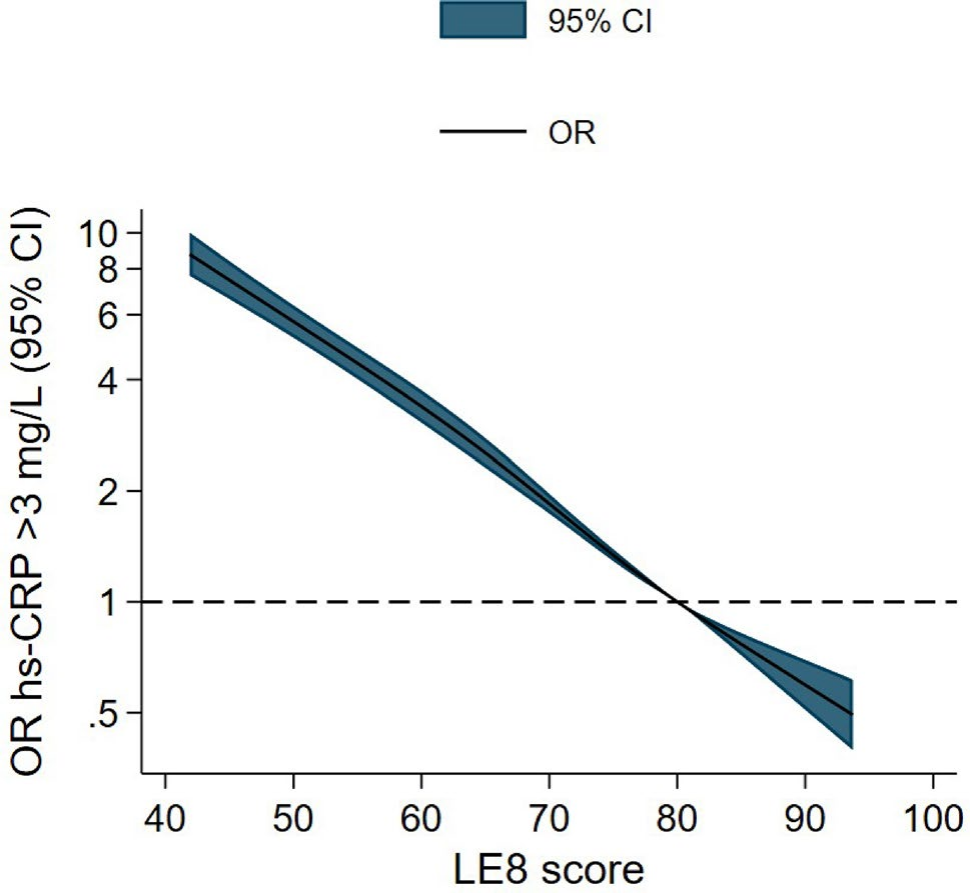
Odds ratios for the association of LE8 score with high-risk hs-CRP. Spline analysis shows adjusted odds ratios (solid line) and 95% confidence intervals (colored area) for high-risk hs-CRP (> 3.0 mg/L) according to total LE8 score. Odds ratios were adjusted for age, sex, study center, education and alcohol use. Reference category was set at 80 points. X-axis was truncated at the 1st and 99th percentile of LE8 values. CI: confidence interval, hs-CRP: high-sensitivity C-reactive protein, LE8: Life’s Essential 8 score, OR: odds ratio.

LE8 scores were strongly, dose–response and inversely associated with high-risk hs-CRP (> 3.0 mg/L), (Fig. 2). For instance, in the spline model adjusted for age, sex, study center, education, and alcohol use, participants with LE8 scores = 50.0 points had 5.8 times greater odds of having high-risk hs-CRP compared to those with LE8 scores = 80.0 points, OR 5.8, 95% CI 5.2–6.4, (Supplementary Table 1).

**Figure 3:**
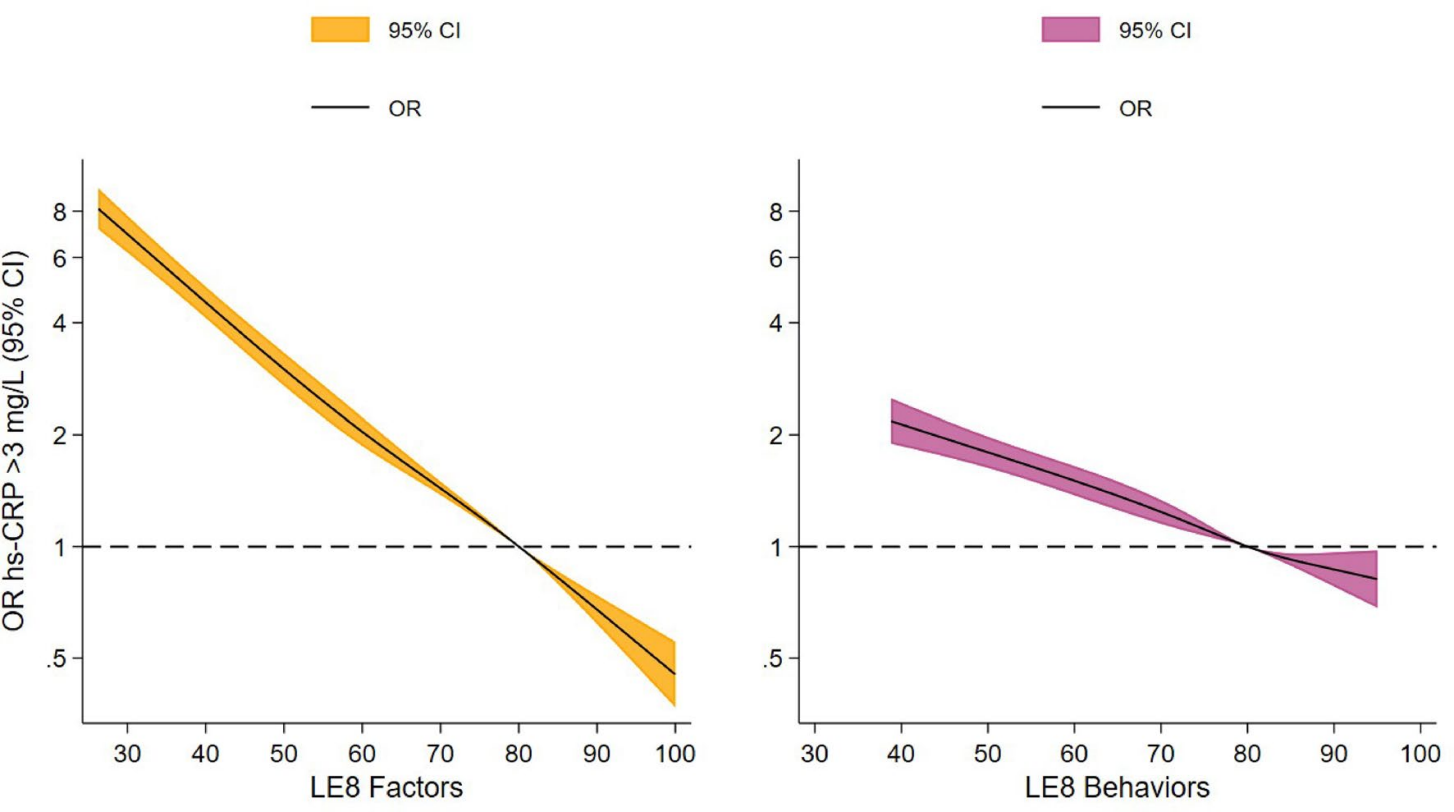
Odds ratios for the associations of LE8 health factors and LE8 health behaviors scores with high-risk hs-CRP. Spline analyses show adjusted odds ratios (solid line) and 95% confidence intervals (colored area) for high-risk hs-CRP (> 3.0 mg/L) according to LE8 health factors and LE8 health behaviors scores. Odds ratios were adjusted for age, sex, study center, education and alcohol use. Reference categories were set at 80 points. X-axes were truncated at the 1st and 99th percentile of LE8 values. CI: confidence interval, hs-CRP: high-sensitivity C-reactive protein, LE8: Life’s Essential 8 score, OR: odds ratio.

When LE8 was divided according to health behaviors and health factors, the inverse association with hs-CRP remained, finding somewhat weaker associations for health behaviors compared to health factors. For instance, in the model considering adjusted splines, participants with LE8 health behaviors score = 50.0 points and LE8 health factors score = 50.0 points had adjusted ORs of 1.8 (95% CI 1.6–2.0) and 3.0 (95% CI 2.7–3.3) for high-risk hs-CRP, respectively, compared to those with a LE8 score = 80.0 points (Supplementary Table 1, Fig. 3). Supplementary Table 2 depicts the association between LE8 categories (low, intermediate and high) and high-risk hs-CRP. Thus, participants with low (≤ 49 points) and intermediate CVH (50–79 points) LE8 score had 8.8- and 3.0-fold increase in the odds for high-risk hs-CRP compared to participants with high LE8 score (≥ 80 points).

In the analysis considering individual components of LE8 (diet, physical activity, nicotine exposure, sleep duration, BMI, non-HDL cholesterol, blood glucose and blood pressure), all 8 components, and particularly BMI, were significantly associated with high-risk hs-CRP (Supplementary Table 3). Thus, in the adjusted model, each standard deviation increase in the BMI LE8 score was associated with 52% reduction in the odds of high-risk hs-CRP (OR = 0.48 [95% CI 0.47–0.50]).

Finally, there was a statistically significant interaction between LE8 and sex in relation to hs-CRP, (p < 0.001), with women exhibiting slightly stronger association of LE8 with hs-CRP than men (Supplementary Fig. 1).

### Sensitivity analyses

Sensitivity analyses using a more conservative physical activity cut-off revealed that the association between LE8 and high-risk hs-CRP remains similar, even when the amount of required moderate-vigorous physical activity to achieve an ideal score was doubled compared to the main analyses (Supplementary Table 4). In addition, the association between LE8 and hs-CRP remained when incorporating additional adjustments for economic status and history of cardiovascular disease (Supplementary Table 5) and when also including participants (n = 665) with hs-CRP > 10 mg/L (Supplementary Table 6).

## Discussion

This large population-based study showed strong and inverse associations between LE8 scores and high-risk hs-CRP. Furthermore, there were inverse associations between LE8 behaviors and factors in relation to high-risk hs-CRP, although the association appeared to be stronger for LE8 factors. Finally, all eight components in the LE8 concept showed a similar inverse association with high-risk hs-CRP.

To the best of our knowledge, the current study is the first investigating the association between the new CVH score LE8 and hs-CRP, which complicates comparison with previous studies. However, an inverse relationship between the previous AHA cardiovascular score, LS7, and hs-CRP has been shown in previous studies^17–22^. These previous studies have mainly been conducted in China and the United States^18–22^. Data from other populations are of interest, considering that hs-CRP levels differ according to socioeconomic and ethnic factors^28^.

It is well-known that inflammation plays a central role in cardiovascular diseases^1–3^. An association between low CVH and inflammation may further reveal the mechanisms of cardiovascular diseases. In our study, all the individual components of LE8 had an association with inflammation which is supported by previous research. It is well-known that smoking, through oxidative stress, and adipose tissue promotes systemic inflammation^29^. Diet may directly promote inflammation as well as cause growth of adipose tissue^30^. Sleep deprivation is also thought to raise inflammation and thereby cause progression of atherosclerosis^31^. In opposite, physical activity leads to increased insulin sensitivity which in turn reduces systemic inflammation^29^. Hyperglycemia and hyperlipidemia affect nitric oxide levels causing endothelial dysfunction which may increase hs-CRP levels^32^. Hypertension is associated with inflammation although the mechanisms are not fully understood^33^. These described drivers and inhibitors of inflammation may be part of the explanation of the inverse association between high estimated CVH by LE8 and hs-CRP levels found in this study. However, the relationship between CVH and hs-CRP is not fully understood, and the role of subclinical inflammation needs further exploration. Meanwhile, measurement of hs-CRP may be used clinically as a simple and easily accessible tool in refined assessments of cardiovascular risk in patients^34^. The European Society of Cardiology suggests that hs-CRP may be helpful for refining CVD risk assessment in patients^34^. In consonance with the European Society of Cardiology, hs-CRP is also listed as a risk enhancer by the American College of Cardiology and the AHA in the guideline on the primary prevention of cardiovascular disease^35^. However, a recent review suggested that, although CRP is a useful biomarker in cardiovascular risk assessment, CRP may not be a likely cause of cardiovascular disease or a therapeutic target^36^. Nevertheless, it has been shown that statins, lipid-lowering agents, reduce hs-CRP levels and the incidence of major cardiovascular events in healthy individuals with elevated hs-CRP but without hyperlipidemia^37^.

The burden of cardiovascular diseases on public health and health care is significant and the reduction of cardiovascular diseases have great financial implications as well as importance to individuals’ health and quality of life. The individual components of LE8 as well as health behaviors and factors grouped together are strongly associated with sub-clinical inflammation, and our findings further support the promotion of health behaviors and factors. Promoting LE8, which consists of eight modifiable variables, may reduce low-grade inflammation. However, further studies are needed to determine through which mechanisms the association is mediated, and the mechanisms connecting LE8 and hs-CRP.

This study has several strengths which include the large sample size with equal proportion of men and women, low rate of missingness and the comprehensiveness of the LE8 score which includes eight important variables. The key strength of our study is the randomized sampling from the Swedish population register. The study also has limitations that should be acknowledged. First, inferring causality between high LE8 scores and hs-CRP is not possible due to the cross-sectional design of the study. Second, the CVH metrics smoking, sleep and diet are assessed by self-reported questionnaires which may have caused recall bias. In addition, although the use of accelerometry is considered a gold standard method for physical activity, there could be a slight overestimation of the levels of physical activity in SCAPIS^38^. However, similar conclusions remain when considering a sensitivity analysis with a more conservative cutoff for the categorization of the LE8 physical activity component. Also, the age of our participants makes generalization to the younger and older population uncertain. Lastly, the generalizability of our study is also limited by, although expected, the lower participation rates in less affluent areas (37% vs. 67%)^23^. Thus, further studies of LE8 and hs-CRP in other populations are warranted.

In conclusion, LE8 scores were strongly and inversely associated with high-risk hs-CRP. This indicates less low-grade inflammation in individuals with high LE8 scores. Although further studies are needed, and preferably a randomized controlled trial to study if the promotion of LE8 does in fact lead to the decrease of subclinical inflammation, our findings suggest that health behaviors as well as health factors are important in the reduction of subclinical inflammation.

### Supplementary Information

Supplementary Information.

## Data Availability

The data analyzed in this study cannot be shared publicly due to legal reasons and the privacy of the study participants. However, by contacting the study organization (http://www.scapis.org) or the corresponding author, information will be provided regarding the procedures for accessing data following Swedish legislation. Requests to access these datasets should be directed to pontus.henriksson@liu.se.
