# Supplementary material for: Life’s Essential 8 is inversely associated with high-sensitivity C-reactive protein
Source: Sci Rep. 2024 Jul 1;14:15024. doi: 10.1038/s41598-024-65977-3 (PMC11217377; doi:10.1038/s41598-024-65977-3)
Supplement: Supplementary file 1 — Supplementary Information. [file 41598_2024_65977_MOESM1_ESM.pdf]

Life's Essential 8 is inversely associated with high-sensitivity C-reactive protein

Lana Hebib[1], Angel Herraiz-Adillo[1], Sara Higueras-Fresnillo[1-2], Daniel Berglind[3-4], Bledar Daka[5], Patrik Wennberg[6], Emil Hagström[7], Cecilia Lenander[8], Viktor H. Ahlqvist[9-10], Carl Johan Östgren[1,11], Karin Rådholm*[1,12], Pontus Henriksson*[1]

[1] Department of Health, Medicine and Caring Sciences, Linköping University, Linköping, Sweden.

[2] Department Physical Education, Sport and Human Motricity, Universidad Autónoma de Madrid, Spain.

[3] Department of Global Public Health, Karolinska Institutet, Stockholm, Sweden.

[4] Centre for Epidemiology and Community Medicine, Region Stockholm, Stockholm, Sweden.

[5] School of Public Health and Community Medicine, Institute of Medicine, Sahlgrenska Academy, University of Gothenburg, Gothenburg, Sweden.

[6] Department of Public Health and Clinical Medicine, Umeå University, Umeå, Sweden.

[7] Department of Medical Sciences, Cardiology, Uppsala University, Uppsala, Sweden.

[8] Department of Clinical Sciences in Malmö, Centre for Primary Health Care Research, Lund University, Lund, Sweden.

[9] Department of Biomedicine, Aarhus University, Aarhus, Denmark.

[10] Institute of Environmental Medicine, Karolinska Institutet, Stockholm, Sweden.

[11] Centre for Medical Image Science and Visualization (CMIV), Linköping university, Linköping, Sweden.

[12] The George Institute for Global Health, University of New South Wales, Sydney, Australia.

*These authors contributed equally to this article and are joint senior authors.

<u>Supplementary material</u>

Supplementary Table 1. Odds ratios for the continuous (splines) associations of LE8 global, LE8 health behaviors and LE8 health factors scores with high-risk hs-CRP

Supplementary Table 2. Odds ratios for the categorical associations of LE8 global, LE8 health behaviors and LE8 health factors scores with high-risk hs-CRP

Supplementary Table 3. Odds ratios for the associations of LE8 health behaviors and LE8 health factors Z scores with high-risk hs-CRP

Supplementary Table 4. Sensitivity analysis, odds ratios for the associations of global LE8 score and LE8 health behavior score with high-risk hs-CRP considering a conservative cut-off for physical activity (300 minutes of moderate or greater intensity of physical activity per week)

Supplementary Table 5. Sensitivity analysis, odds ratios for the associations of global LE8 score with high-risk hs-CRP considering additional adjustments for economic status and history of cardiovascular disease

Supplementary Table 6. Sensitivity analysis, odds ratios for the association of global LE8 score with high-risk hs-CRP including participants (n=665) with hs-CRP >10 mg/L

Supplementary Figure 1. Odds ratios for the association of LE8 scores with high-risk hs-CRP by sex

Supplementary Table 1. Odds ratios for the continuous (splines) associations of LE8 global, LE8 health behaviors and LE8 health factors scores with high-risk hs-CRP

	**Unadjusted**	**Adjusted**[1]
**LE8 global**	**OR (95% CI)**	**OR (95% CI)**
30	17.04 (13.88-20.93)	16.25 (13.14-20.09)
40	9.88 (8.60-11.36)	9.67 (8.36-11.18)
50	5.73 (5.21-6.31)	5.75 (5.20-6.36)
60	3.30 (2.99-3.63)	3.38 (3.06-3.74)
70	1.80 (1.70-1.91)	1.84 (1.74-1.96)
80	Reference	Reference
90	0.61 (0.52-0.71)	0.60 (0.51-0.69)
100	0.38 (0.27-0.53)	0.36 (0.26-0.50)
**LE8 health behaviors**	**OR (95 % CI)**	**OR (95 % CI)**
30	3.16 (2.63-3.79)	2.53 (2.10-3.06)
40	2.54 (2.24-2.89)	2.13 (1.87-2.44)
50	2.05 (1.87-2.25)	1.79 (1.63-1.98)
60	1.64 (1.50-1.79)	1.50 (1.37-1.65)
70	1.29 (1.20-1.39)	1.24 (1.15-1.33)
80	Reference	Reference
90	0.87 (0.78-0.97)	0.87 (0.78-0.96)
100	0.79 (0.61-1.01)	0.77 (0.60-0.99)
**LE8 health factors**	**OR (95 % CI)**	**OR (95 % CI)**
30	6.61 (5.93-7.37)	6.93 (6.19-7.77)
40	4.32 (3.94-4.74)	4.54 (4.12-5.00)
50	2.86 (2.59-3.15)	3.00 (2.71-3.32)
60	1.96 (1.80-2.13)	2.04 (1.87-2.22)
70	1.41 (1.35-1.46)	1.44 (1.38-1.49)
80	Reference	Reference
90	0.69 (0.63-0.75)	0.68 (0.62-0.74)
100	0.47 (0.38-0.57)	0.45 (0.37-0.55)

[1] Odds ratios were adjusted for age, sex, study center, education, and alcohol use.
CI: confidence interval, CVH: cardiovascular health status, hs-CRP: high-sensitivity C-reactive protein, LE8: Life’s Essential 8, OR: odds ratio.

Supplementary Table 2. Odds ratios for the categorical associations of LE8 global, LE8 health behaviors and LE8 health factors scores with high-risk hs-CRP

	**Unadjusted**	**Adjusted**[1]
**LE8 global**	**OR (95% CI)**	**OR (95% CI)**
≤49	9.11 (7.80-10.66)	8.80 (7.47-10.37)
50-79	3.00 (2.69-3.35)	3.03 (2.71-3.40)
≥80	Reference	Reference
**LE8 health behaviors**	**OR (95% CI)**	**OR (95% CI)**
≤49	2.65 (2.32-3.02)	2.34 (2.04-2.68)
50-79	1.55 (1.44-1.67)	1.49 (1.38-1.60)
≥80	Reference	Reference
**LE8 health factors**	**OR (95% CI)**	**OR (95% CI)**
≤49	6.00 (5.37-6.70)	6.46 (5.75-7.26)
50-79	2.39 (2.16-2.65)	2.50 (2.25-2.78)
≥80	Reference	Reference

[1] Odds ratios were adjusted for age, sex, study center, education and alcohol use.
CI: confidence interval, CVH: cardiovascular health status, hs-CRP: high-sensitivity C-reactive protein, LE8: Life’s Essential 8, OR: odds ratio.

Supplementary Table 3. Odds ratios for the associations of LE8 health behaviors and LE8 health factors Z scores with high-risk hs-CRP

	Unadjusted		Adjusted[1]	
	**OR (95% CI)**	**P**	**OR (95% CI)**	**P**
**LE8 health behaviors**				
Diet	0.90 (0.87-0.93)	<0.001	0.88 (0.85-0.91)	<0.001
Physical activity	0.82 (0.80-0.84)	<0.001	0.84 (0.82-0.86)	<0.001
Smoking	0.82 (0.80-0.85)	<0.001	0.86 (0.81-0.89)	<0.001
Sleep	0.87 (0.85-0.90)	<0.001	0.90 (0.87-0.93)	<0.001
**LE8 health factors**				
BMI	0.48 (0.46-0.49)	<0.001	0.48 (0.47-0.50)	<0.001
Non-HDL cholesterol	0.83 (0.81-0.86)	<0.001	0.84 (0.81-0.87)	<0.001
Blood glucose	0.70 (0.68-0.73)	<0.001	0.70 (0.68-0.73)	<0.001
Blood pressure	0.73 (0.70-0.75)	<0.001	0.71 (0.69-0.74)	<0.001

LE8 health behaviors and LE8 health factors are summarized as Z scores (mean=0, SD=1).
[1] Odds ratios were adjusted for age, sex, study center, education level, and alcohol use.
CI: confidence intervals, CVH: cardiovascular health, HDL: high-density lipoprotein, hs-CRP: high-sensitivity C-reactive protein, LE8: Life’s Essential 8, OR: odds ratio, SD: standard deviation.

Supplementary Table 4. Sensitivity analysis, odds ratios for the associations of global LE8 score and LE8 health behavior score with high-risk hs-CRP considering a conservative cut-off for physical activity (300 minutes of moderate or greater intensity of physical activity per week)

	**OR[1] (95% CI)**	**P**
**LE8 global**		
≤49	9.48 (8.12-11.05)	<0.001
50-79	2.93 (2.60-3.29)	<0.001
≥80	Reference	
**LE8 health behaviors**		
≤49	2.67 (2.37-3.02)	<0.001
50-79	1.52 (1.41-1.64)	<0.001
≥80	Reference	

[1] Odds ratios were adjusted for age, sex, study center, education level and alcohol use.
CI: confidence intervals, hs-CRP: high-sensitivity C-reactive protein, LE8: Life's Essential 8, OR: odds ratio.

Supplementary Table 5. Sensitivity analysis, odds ratios for the associations of global LE8 score with high-risk hs-CRP considering additional adjustments for economic status and history of cardiovascular disease

**Main analysis**		
**LE8 global score**	**OR[1] (95% CI)**	**P**
≤49	8.80 (7.47-10.37)	<0.001
50-79	3.03 (2.71-3.40)	<0.001
≥80	Reference	
**Main analysis + economic status**		
**LE8 global score**	**OR[2] (95% CI)**	**P**
≤49	8.05 (6.81-9.52)	<0.001
50-79	2.91 (2.60-3.26)	<0.001
≥80	Reference	
**Main analysis + history of cardiovascular diseases status**		
**LE8 global score**	**OR[3] (95% CI)**	**P**
≤49	8.60 (7.28-10.16)	<0.001
50-79	2.97 (2.66-3.33)	<0.001
≥80	Reference	

[1] Odds ratios were adjusted for age, sex, study center, education level and alcohol use.
[2]Additional adjustment for economic status.
[3]Additional adjustment for history of cardiovascular disease (self-reported myocardial infarction, coronary artery bypass grafting, percutaneous coronary intervention, stroke, or peripheral arterial disease intervention).
CI: confidence intervals, hs-CRP: high-sensitivity C-reactive protein, LE8: Life’s Essential 8, OR: odds ratio.

Supplementary Table 6. Sensitivity analysis, odds ratios for the association of global LE8 score with high-risk hs-CRP including participants (n=665) with hs-CRP >10 mg/L

**LE8 global**	**OR[1] (95% CI)**
≤49	8.38 (7.17-9.79)
50-79	2.84 (2.56-3.14)
≥80	Reference
**LE8 health behaviors**	**OR (95% CI)**
≤49	2.28 (1.99-2.60)
50-79	1.43 (1.33-1.53)
≥80	Reference
**LE8 health factors**	**OR (95% CI)**
≤49	5.89 (5.28-6.56)
50-79	2.33 (2.12-2.57)
≥80	Reference

[1] Odds ratios were adjusted for age, sex, study center, education and alcohol use.
CI: confidence interval, CVH: cardiovascular health status, hs-CRP: high-sensitivity C-reactive protein, LE8: Life’s Essential 8, OR: odds ratio.

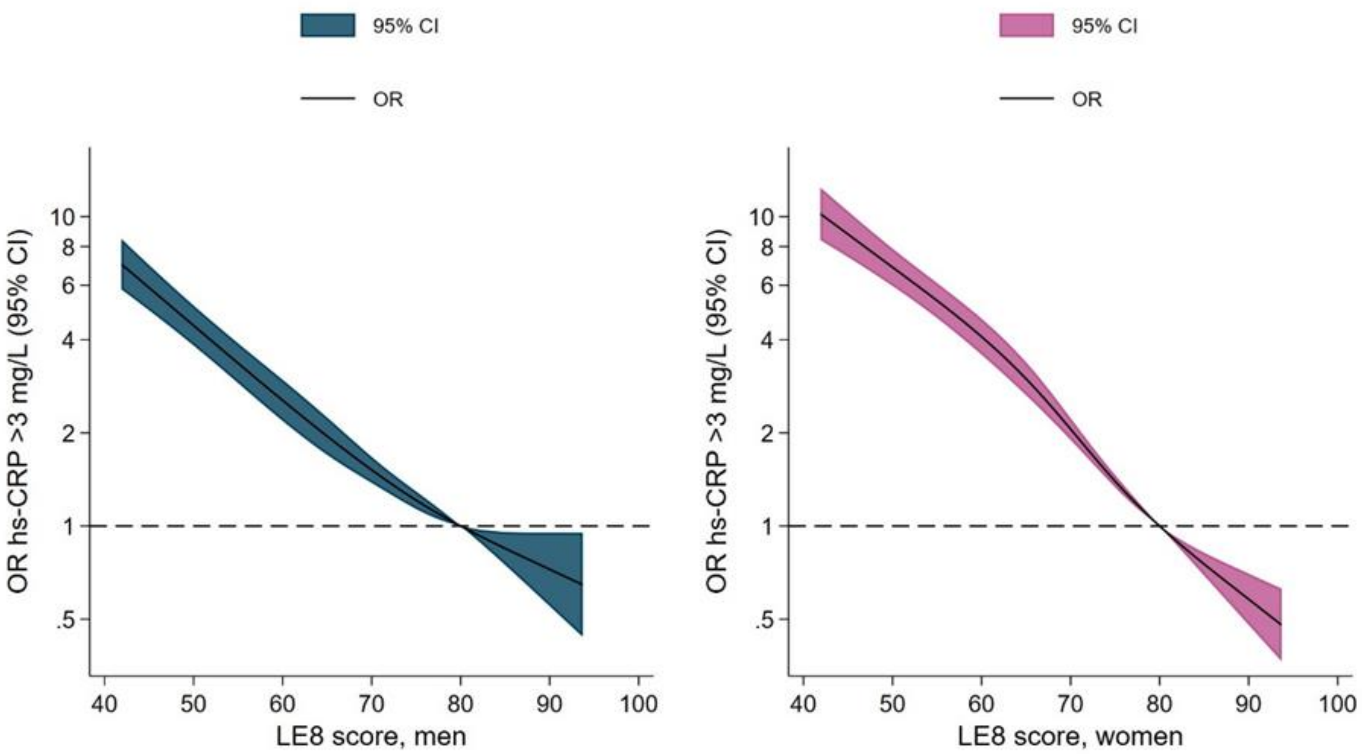

Supplementary Figure 1. Odds ratios for the association of LE8 scores with high-risk hs-CRP by sex.

Spline analyses show adjusted odds ratios (solid line) and 95% confidence intervals (colored area) for high-risk hs-CRP (>3.0 mg/L) according to LE8 scores.

Odds ratios were adjusted for age, study center, education and alcohol use.

Reference categories were set at 80 points. X-axes were truncated at the 1st and 99th percentile of LE8 values.

CI: confidence interval, hs-CRP: high-sensitivity C-reactive protein, LE8: Life’s Essential 8 score, OR: odds ratio.
